# Medical implications of technical accuracy in genome sequencing

**DOI:** 10.1186/s13073-016-0269-0

**Published:** 2016-03-02

**Authors:** Rachel L. Goldfeder, James R. Priest, Justin M. Zook, Megan E. Grove, Daryl Waggott, Matthew T. Wheeler, Marc Salit, Euan A. Ashley

**Affiliations:** Department of Medicine, Stanford University, Stanford, CA 94305 USA; Genome-scale Measurements Group, National Institute of Standards and Technology, Gaithersburg, MD 20899 USA; Stanford Center for Inherited Cardiovascular Disease, Stanford University, Stanford, CA 94305 USA; Department of Genetics, Stanford University, Stanford, CA 94305 USA; Department of Pediatrics, Stanford University, Stanford, CA 94305 USA

## Abstract

**Background:**

As whole exome sequencing (WES) and whole genome sequencing (WGS) transition from research tools to clinical diagnostic tests, it is increasingly critical for sequencing methods and analysis pipelines to be technically accurate. The Genome in a Bottle Consortium has recently published a set of benchmark SNV, indel, and homozygous reference genotypes for the pilot whole genome NIST Reference Material based on the NA12878 genome.

**Methods:**

We examine the relationship between human genome complexity and genes/variants reported to be associated with human disease. Specifically, we map regions of medical relevance to benchmark regions of high or low confidence. We use benchmark data to assess the sensitivity and positive predictive value of two representative sequencing pipelines for specific classes of variation.

**Results:**

We observe that the accuracy of a variant call depends on the genomic region, variant type, and read depth, and varies by analytical pipeline. We find that most false negative WGS calls result from filtering while most false negative WES variants relate to poor coverage. We find that only 74.6 % of the exonic bases in ClinVar and OMIM genes and 82.1 % of the exonic bases in ACMG-reportable genes are found in high-confidence regions. Only 990 genes in the genome are found entirely within high-confidence regions while 593 of 3,300 ClinVar/OMIM genes have less than 50 % of their total exonic base pairs in high-confidence regions. We find greater than 77 % of the pathogenic or likely pathogenic SNVs currently in ClinVar fall within high-confidence regions. We identify sites that are prone to sequencing errors, including thousands present in publicly available variant databases. Finally, we examine the clinical impact of mandatory reporting of secondary findings, highlighting a false positive variant found in *BRCA2*.

**Conclusions:**

Together, these data illustrate the importance of appropriate use and continued improvement of technical benchmarks to ensure accurate and judicious interpretation of next-generation DNA sequencing results in the clinical setting.

**Electronic supplementary material:**

The online version of this article (doi:10.1186/s13073-016-0269-0) contains supplementary material, which is available to authorized users.

## Background

As whole exome sequencing (WES) and whole genome sequencing (WGS) transition from research tools to clinical diagnostic tests, it is increasingly critical for sequencing methods and analysis pipelines to be technically accurate. To interpret appropriately the results of any clinical test, the informed clinician should have a working knowledge of the accuracy and diagnostic characteristics of the test. Initial evaluations suggest that SNV and INDEL genotype calls can vary based on exome capture kit, sequencing platform, and the aligner and variant caller [1–9]. An absence of technical benchmark data and evaluation methods prompted the National Institute of Standards and Technology (NIST) to convene the Genome in a Bottle (GIAB) Consortium to develop infrastructure to address this problem. The consortium is developing and disseminating Reference Materials, Reference Data, and Reference Methods for human genome sequencing.

**Fig. 1:**
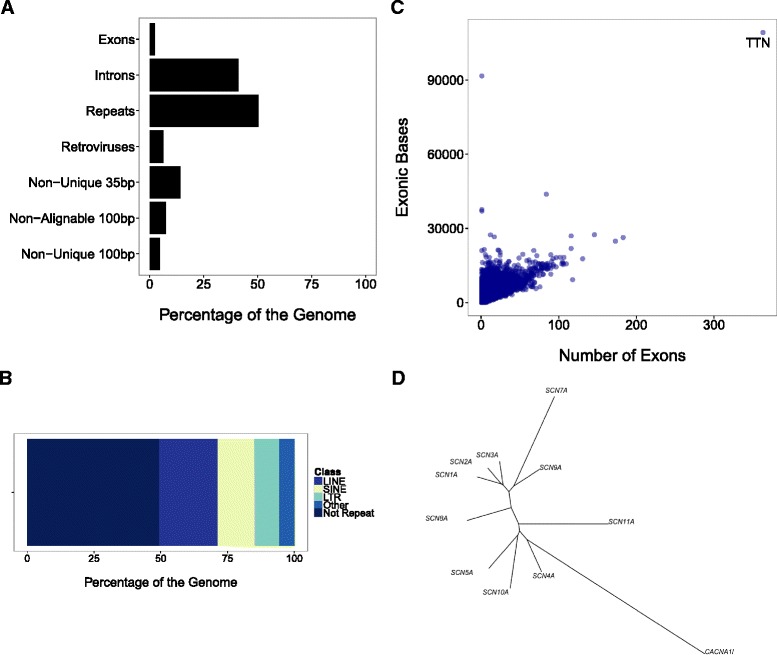
Complexity of the Genome. **a** The genome consists of several (overlapping) regions. Eighty-six percent of 35 bp sequences and 95 % of 100 bp sequences are unique to one location in the reference genome. **b** A total of 50.6 % of the non-N reference genome falls into a repeat (data from RepeatMasker). **c** There is great variation in exon count and number of exonic bases per gene (data from RefSeq). **d** An unrooted phylogenetic tree derived from multiple alignment of cDNA sequences of 10 voltage-gated sodium channel genes within the human genome illustrates the complexity evolutionary relationship of paralogous sequences which complicates the process of short-read alignment in next-generation sequencing. A related voltage gated calcium channel *CACNA1L* is included as an outgroup

The complex nature of the human genome presents significant challenges to achieving technical accuracy in clinical sequencing (Fig. 1). A human genome contains 3.2 billion basepairs (bp) consisting of 50–69 % repetitive sequence [10] encompassing transposable elements (LINES, SINES, and Long Terminal Repeats), low complexity regions (such as homopolymers), and pseudogenes. Larger insertions, deletions, and rearrangements within the genome, often termed structural variants, are not represented in a reference sequence and thus present additional complexity in alignment. A total of 19,000–21,000 protein coding genes comprise 1–2 % of the genome [11], and the size of protein-coding genes is variable. RefSeq genes have a median of six exons per gene with the titin (*TTN*) containing the highest number of exons: 363. Certain disease-related genes are particularly complex, such as the highly paralogous families of transmembrane ion channels, many of which are associated with cardiac arrhythmias and excitatory abnormalities in the nervous system [12]. The challenges of repetitive, paralogous sequence and structural variation complicate the analysis of clinical WGS and WES data. Not only is short-read sequencing prone to false negative or false positive variant calls due to systematic sequencing errors, but the repetitive nature of the genome introduces global mapping and local alignment challenges [13].

Over the last several years many groups have demonstrated the clinical utility of genome sequencing [14–17], developing tools for clinical interpretation of individuals [18], families [19], and for rapid genetic diagnosis [20–24]. Themes throughout this work include low concordance across platforms for insertion-deletion variants, and moderate concordance between interpreters of genomic variants [1, 5, 25].

In this analysis, we characterize the GIAB [26] high-confidence regions, benchmark WGS and WES example variant calls in relation to publicly available high-confidence consensus SNV, indel, and homozygous reference genotypes for NA12878, and evaluate the clinical impact of genomic sites with systematic errors from one or more sequencing platforms. We use the WGS and WES benchmark to investigate the causes of extra and missing variants in two call sets (putative false positive and false negative variants, respectively). We focus on potentially functionally significant variants. Finally, we compare performance across the whole genome to performance for different types of potentially functional variants in genes that have different levels of evidence for disease association and clinical actionability.

## Methods

### Reference genome, sequencing platforms, and variant calling

We recently published a set of high-confidence SNV, indel, and homozygous reference genotypes for the pilot whole genome NIST Reference Material 8398 [26]. Briefly these genotypes were generated by integrating 14 whole genome and exome sequencing datasets from five different technologies. When the datasets had discordant genotypes, we arbitrated between them using characteristics of bias typically used for filtering variants, such as strand bias, mapping quality, and clipping of reads. Specifically, at sites with discordant genotypes, we used genotypes from datasets that did not have characteristics of bias. If the reason for the discordant genotypes could not be automatically determined using the characteristics of bias (for example, if datasets with no evidence of bias disagreed), then the variant and surrounding region was excluded from the high-confidence regions. Additionally, we excluded regions if all datasets had evidence of bias or fewer than 5 reads with mapping quality >10. We also excluded regions in which current sequencing technologies are prone to errors (specifically, long homopolymers and tandem repeats, segmental duplications, and putative structural variants). The resulting high-confidence calls and high-confidence regions for this pilot genome, based on DNA from subject NA12878, are rapidly being adopted by clinical and research labs to obtain performance metrics such as sensitivity and false discovery rate for new library preparation and informatics methods [3, 27–32].

One whole genome and one Nextera-based whole exome sequencing dataset from the Illumina HiSeq sequencing platform were used in this work. The coverage of coding regions by the Nextera exome kit was found to be better than other standard exome kits, but worse than newer enhanced exome library preparation methods like ‘augmented exome sequencing’ [33]. This sequencing was performed in 2013 and 2014 by two participating institutions of the Genome in a Bottle Consortium: NIST and the Garvan Institute of Medical Research. The sequencing was done on the candidate NIST Reference Material 8398, a large batch of DNA extracted from the cell line GM12878. The cell line is archived at the Coriell Institute for Medical Research. These measurements represent typical approaches that were broadly used at the time of this study.

Whole genome sequencing of 150 × 150 bp paired end reads was performed on the Illumina HiSeq 2500 with PCR-free v2 chemistry at NIST. These data were from 12 flow cells on the same instrument and 14 replicate libraries prepared from a total of six tubes of candidate NIST RM 8398. The raw data were aligned using BWA MEM v.0.7.5a with default parameters [34]. Reads from each library from each lane were independently realigned using GATK v.2.8-1-g932cd3a IndelRealigner, followed by Base Quality Score Recalibration following GATK Best Practices [35]. Then, all reads from all runs and libraries were combined for a second round of GATK IndelRealigner. The reads were randomly downsampled from approximately 300× to 30× coverage to give a typical level of coverage for WGS. Note that this amounted to 31× coverage within the Nextera exome capture regions. Even though these data are from multiple libraries and runs, we expect that these should represent typical data for the purposes of this work, though they may contain slightly fewer errors since errors from any particular library would be diluted by combining with other libraries. Variants were called using Platypus v.0.5.2 including assembly-based calling to test a new pipeline that was recently proposed for clinical variant calling [36]. Variants were filtered using the defaults for Platypus (that is, GOF, badReads, alleleBias, hp10, Q20, HapScore, MQ, strandBias, SC, QualDepth, REFCALL, and QD) [36]. Separately, INDELs were called using Scalpel [37] version 0.3.2 in single sample mode for CCDS regions with default settings. The entire 300× dataset and the 30× downsampled bam file are available on the GIAB ftp site at NCBI: ftp://ftp-trace.ncbi.nlm.nih.gov/giab/ftp/data/NA12878/NIST_NA12878_HG001_HiSeq_300x/.

Approximately 50× coverage whole exome sequencing was performed on a library prepared using the Nextera rapid capture exome kit at the Garvan Institute of Medical Research. The raw read data were aligned using BWA and variants were called using GATK HaplotypeCaller v.2.7-2-g6bda569 [35]. No filtering was applied. Note that the variant calling pipelines for WES differs from that of the WGS. The vcf file used is available on the GIAB ftp site at NCBI: ftp://ftp-trace.ncbi.nlm.nih.gov/giab/ftp/data/NA12878/analysis/GARVAN_snps_indels_12172013/project.NIST.hc.snps.indels.vcf Separately, INDELs were called using Scalpel [37] version 0.3.2 in single sample mode for CCDS regions with default settings.

### Comparison to GIAB benchmark calls

We compared the WGS and WES calls to the latest version of high-confidence calls from GIAB, which integrates multi-platform integrated calls from NIST with two phased pedigree call sets from Real Time Genomics and the Illumina Platinum Genomes Project (from: ftp://ftp-trace.ncbi.nlm.nih.gov/giab/ftp/release/NA12878_HG001/GIABPedigreev0.2/). To compare different representations of complex variants (that is, nearby SNVs and/or indels), we used the freely available Real Time Genomics tool vcfeval (ftp://ftp-trace.ncbi.nlm.nih.gov/giab/ftp/tools/RTG/). The resulting calls in the test sets that were included (true positives), extra (false positives), and missing (false negatives) in the benchmark were then annotated for potential functional effect.

### Annotation and variant classification

We annotated variant call sets with Sequence to Medical Phenotypes (STMP), which employs a custom Annovar-based tool to integrate data into a tabular format from 94 sources, including segmental duplications, repetitive elements, ClinVar and OMIM annotations, and performs separate functional annotations with transcript information from NCBI RefSeq, Ensembl, and UCSC [1, 38]. The data were further sorted and variants tabulated with custom python scripts. Individual variants were manually curated for technical validity (JZ) and potential clinical relevance (MG).

### Gene sets

We define two gene sets. The American College of Medical Genetics and Genomics (ACMG) reportable genes list contains the 56 genes that the ACMG recommend for pathogenic variant discovery and reporting [39]. Though it contains only a fraction of important disease-related genes, we selected the list because it represents an externally defined minimal set of genes where performance must meet clinical standards. It also represents a group of genes felt to be medically actionable, a group where we would hope for optimal technical performance. The second gene set contains genes derived from the ClinVar and OMIM catalogs to represent a total of 3,300 genes with known relationship to human disease.

### Genomic regions

The 35 bp uniqueness scores and 100 bp alignability data were downloaded from the UCSC Genome Browser. The Z bp uniqueness metric indicates whether the sequence (of length-Z) beginning at that base is unique in the genome, while the 100 bp ‘alignability’ metric tolerates up to two mismatches.

The 100 bp uniqueness scores were created by breaking the reference into 100 bp fragments and aligning with Bowtie (allowing no gaps and only accepting unique alignments, options: −v0 –best –m1).

### Sites with systematic errors in relevant databases

We defined sites with systematic errors as sites that were first determined to be homozygous reference by the Genome in a Bottle arbitration process, and second, a non-homozygous reference genotype was called from any sequencing platforms that had reads containing a variant at the site. Specifically, we considered a site to have a systematic error if all sequencing datasets from a platform had evidence for an incorrect genotype or if more than two sequencing datasets from a platform had evidence for an incorrect genotype. No filtering was performed and all variants with a quality score >2 were called using GATK v2.8-1. A low quality score threshold was used to be more comprehensive in finding sites that might have bias. These sites can be downloaded from the GIAB ftp site (ftp://ftp-trace.ncbi.nlm.nih.gov/giab/ftp/data/NA12878/analysis/NIST_union_callsets_06172013/NISTIntegratedCalls_14datasets_131103_allcall_UGHapMerge_HomRef_VQSRv2.18_all_bias_nouncert_excludesimplerep_excludesegdups_excludedecoy_excludeRepSeqSTRs_noCNVs.vcf.gz), and the platform or platforms with systematic errors are listed in the INFO field, ‘platformbias’. We used bedtools [40] to intersect the coordinates of these variants with those in annotation databases and custom perl scripts to filter out variants in annotation databases with different alternative alleles.

### Characterizing the GIAB high-confidence regions

We restricted benchmarking to regions of the Genome in a Bottle reference material determined to be high confidence. As described above and in our previous work [26], we excluded regions from the high-confidence regions if they were low coverage, prone to mapping error (paralogous sequences, repetitive elements, structural variants, and segmental duplications) or systematic errors in all sequencing chemistries (repetitive elements, low-complexity regions). We characterized the high-confidence regions in terms of uniqueness, repeat sequences, and the proportion of the genome and exome that fall inside these high-confidence regions.

## Results

### Accuracy of variant calls in high-confidence regions

In the high-confidence regions, we assessed the accuracy of variant calls from Illumina whole genome (BWA MEM followed by Platypus) and Illumina Nextera exome sequencing (BWA followed by GATK).

We compared the performance for different types of potentially functional SNVs in medically relevant genes from ClinVar/OMIM as well as genome wide. For all functional annotations (non-synonymous, synonymous, splicing, and truncating) WGS enabled equal or higher average sensitivity compared to WES (Table 1). Similarly, for INDELs, sensitivity was higher for WGS (72.7 %) than WES (22.7 %) within consensus coding and high-confidence regions using Scalpel [37].

**Table 1:** Sensitivities for whole genome sequencing (WGS) and whole exome sequencing (WES) SNVs

Function	Gene set	WGS SNV sensitivity	WES SNV sensitivity
Non-synonymous	ClinVarOMIM	0.979 (0.970,0.985)	0.936 (0.923,0.948)
Non-synonymous	Exome	0.979 (0.975,0.982)	0.936 (0.930,0.942)
Splicing	ClinVarOMIM	0.889 (0.565,0.994)	0.556 (0.267,0.811)
Splicing	Exome	0.951 (0.865,0.983)	0.629 (0.505,0.738)
Synonymous	ClinVarOMIM	0.988 (0.982,0.992)	0.952 (0.942,0.961)
Synonymous	Exome	0.985 (0.983,0.988)	0.952 (0.947,0.956)
Truncating	ClinVarOMIM	1.000 (0.646,1.000)	1.000 (0.646,1.000)
Truncating	Exome	1.000 (0.924,1.000)	0.915 (0.801,0.966)
Whole genome	N/A	0.954 (0.954,0.955)	0.053 (0.053,0.053)

Sensitivity for different categories of potentially functional variants across different gene categories. Parentheses contain 95 % binomial confidence intervals

False negatives and false positives arise for different reasons in each platform. For WES, poor read depth was the primary driver of sensitivity as 95 % of false negative variants (FNVs) fell within regions having a read coverage of <10. Note that variant calls remained consistent with increased overall coverage (Additional file 1: Table S1). Further analysis of FNVs revealed that 16 % of whole genome FNVs fall inside simple repeats, low complexity regions, or satellite repeats, compared to 8.6 % of whole exome FNVs. In contrast, 16 % of whole exome FNVs are in regions with GC content >75 %, compared to <1 % of whole genome FNVs.

For WGS, most FNVs resulted from filtering by Platypus due to their presence within difficult-to-sequence and/or difficult-to-call regions. Specifically, 87 % of FNVs were called but removed by filtering using the default parameterization of Platypus (that is, low base qualities, allele frequency, homopolymers >10 bp, variant quality <20, too many haplotypes, low mapping quality, strand bias, low complexity regions). Thirty-six percent of whole genome FNVs fell within the short interspersed nuclear elements (SINE) class of repetitive elements, compared to 13 % of all whole genome bases residing within SINEs, and less than 1 % of FNVs within genic SINEs. Since most FNVs for WGS calls in the whole genome were caused by filtering, we characterized which filters were most and least specific in distinguishing likely false positives from likely true positives (Additional file 2: Table S2). The least specific filters for Platypus were haplotype score (HapScore), mapping quality (MQ), sequence context (SC), and QUAL by depth (QD). When these were the only filters, they contained only 3 % to 5 % false positives and together made up 77 % of the FNVs. In contrast, strandBias, a filter indicating that a significantly higher proportion of variants falls on one sequencing strand compared to the other, contained 68 % false positives when it occurred on its own or 99 % false positives when it occurred in addition to another filter. In general, sites with multiple reasons for filtering had a higher false positive rate (39 %) than all filtered sites (15 %). These results highlight the importance of characterizing and tuning filters to obtain the most accurate and complete call-set possible [41].

Less than 3 % of variants in both whole genome and exome sequencing were absent from benchmark calls but fell within high-confidence regions; therefore, one could consider these variants false positives. However, manual inspection of alignments around these variants suggests a variety of etiologies, so we instead call them questionable variants (QVs). For WES, most QVs were correctly identified as non-reference but had incorrect genotypes due to insufficient coverage (for example, the sites were identified as homozygous variant when they were in fact heterozygous). Since exome variant calls were unfiltered, there were also a few QVs that were likely to be systematic sequencing errors; these had clear evidence of strand bias and would be easily filtered if a strand bias filter were applied, including a variant rs200691513 (K856N) in the clinically-relevant, ACMG gene *DSG2*, which is associated with arrhythmogenic right ventricular cardiomyopathy. For WGS, almost all of the QVs represent difficulties in our simple classification schema, in that many likely represent true variants occurring near the boundary between high-confidence and low-confidence regions. In fact, except for a series of seven QVs in *SERPINA1* (discussed below), all six of the remaining synonymous and non-synonymous QVs in ClinVar/OMIM genes were within 50 bp of the inside edge of high-confidence regions. Complex variants are occasionally missing from the high-confidence calls as they overlap the borders of the high and low confidence delineation. Therefore, we recommend manual inspection of QVs near the edge of high-confidence regions.

In one particular region, appropriate alignments and variant calls against the hg19 reference yielded a series of five synonymous and two non-synonymous phased heterozygous QVs between chr14:94844936-94844975 in the gene, *SERPINA1* (Additional file 3: Figure S1a). As shown in Additional file 3: Figure S1b, this gene resides within a larger region that has a curation issue from the Genome Reference Consortium (GRC Curation Issue HG-1930, http://www.ncbi.nlm.nih.gov/projects/genome/assembly/grc/human/issues/?id=HG-1930). These variants are contained in a new alternative sequence that is part of GRCh38 constructed from the 1000 Genomes decoy reference sequence. The GeT-RM browser allows a BLAST search of the sequence in a region, revealing the same series of SNVs in the homologous sequence (Additional file 3: Figure S1c). These seven variants were classified as QVs, because they come from an alternate locus that is unlocalized in the reference assembly. This result highlights that future work is needed to further understand how alternate loci in GRCh38 will be employed in variant calling pipelines to minimize the types of errors classified as QVs and FNVs in our analysis.

### Sites prone to systematic errors may have clinical relevance

Short-read sequencing technologies and analysis pipelines are prone to systematic errors at some genomic locations. These systematic errors may result from PCR amplification, errors sequencing particular sequence contexts, local alignment errors, and/or global mapping errors. We identified 39,301 loci where the benchmark data contain a high-confidence homozygous reference call, but at least one sequencing technology incorrectly called a variant. For this analysis, we define these positions as sites with systematic errors. Strikingly, 7,467 of these variants are present in one or more of the following databases: ClinVar, ESP, 1000 Genomes, COSMIC, and dbSNP (Table 2). These variants in publicly available databases may arise from two sources: they may be false positives that were submitted to the databases, or they may be real variants in the population that are not present in the NA12878 genome. In the first case, systematic sequencing errors interpreted as true positives may detrimentally affect algorithms that use these databases, such as GATK Base Quality Score Recalibration. In the second case, it may be difficult to distinguish between real variants and systematic sequencing errors at these positions in any individual. Of note, only four sites with systematic errors were in ClinVar, all of which were indels in homopolymers from Ion Torrent sequencing experiments, and would likely be filtered by Ion Torrent Variant Caller, which calls more accurately in such contexts (Additional file 4: Table S3). These four sites appeared likely to represent real, disease-associated variants previously reported in other individuals, including a truncating variant in *BRCA2* discussed below.

**Table 2:** Sites with falsely-called variants in one or more technologies and their presence in several databases

	Sites (n)
Total variants with bias	39,301
Total variants with bias in databases	7,467
ClinVar	4
ESP	38
1000 Genomes	89
dbSNP (v138)	7,363
COSMIC	123

### Large areas of medically actionable genes fall within low confidence regions

**Fig. 2:**
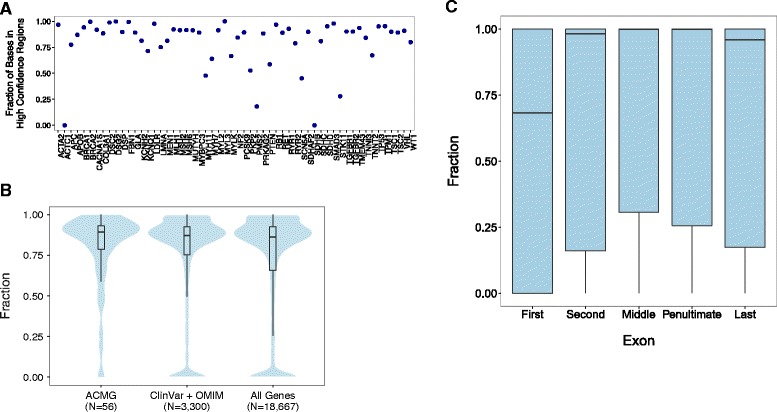
**a** The fraction of each ACMG gene within GIAB high-confidence regions. **b** Violin plots showing the distribution of the fraction each gene in the GIAB high-confidence regions for NA12878 for relevant gene sets: ACMG reportable genes, genes with variants in OMIM or ClinVar, and all genes. **c** Boxplots showing the distribution of the fraction of first, second, middle, penultimate, and last exon in ClinVar or OMIM genes in the GIAB high-confidence regions

We sought to characterize the high-confidence regions in the context of clinical applications. Towards this goal, we calculated the proportion of exonic bases present in the high-confidence regions for each ACMG gene (Fig. 2a). In total, 82.1 % of exonic bases in ACMG genes are in high confidence regions. Individual genes ranged from 0 % to 100 % of exonic bases in high-confidence regions. Table 3 displays the reason for low confident bases in ACMG genes. The most common reasons for low confidence were overlapping with STRs or segmental duplications found in previous studies [42] or purported structural variants in dbVar from NA12878.

**Table 3:** Reasons for low confident bases in ACMG genes

Reason for low confidence	Percentage of bases
CNVs or other SVs that have been reported in dbVar for NA12878	47
STRs in RepSeqSTRdb	34
Regions with known segmental duplications	15
Simple Repeats from repeat masker	1.7
<3 datasets have at least 5 reads with mapping quality >10	1.3
Abnormal allele balance	0.17
Unresolved conflicting genotypes after arbitration	0.03
Calls with support from <3 datasets after arbitration	0.0082
Local alignment problems	0.0041

Next, we calculated the proportion of exonic bases present in the high-confidence regions for ClinVar and OMIM genes and all coding genes (Fig. 2b). Surprisingly, only 74.6 % of ClinVar and OMIM genes’ exonic bases and 72.7 % of the exonic bases in all coding genes are found in high-confidence regions. Of the 18,667 coding genes, 990 were 100 % within high-confidence regions; these genes tend to be smaller (mean: 1,787 bp) than the rest (mean: 3,371 bp).

Large portions of clinically important genes fall outside of the high-confidence regions. A total of 593 of 3,300 ClinVar and OMIM genes have less than 50 % of the exonic bases in high-confidence regions and 2,616 of 18,667 coding genes’ exonic bases are entirely excluded from high-confidence regions.

We also examined ClinVar and OMIM genes at the exon-level; Fig. 2c shows the distribution of the proportion of first, second, middle, penultimate, and last exons inside the high-confidence regions. Notably, first exons have a lower than average proportion of their bases in high-confidence regions, which is likely explained by the well-known higher GC content in first exons.

### High-confidence regions are enriched for unique and non-repetitive sequences

**Fig. 3:**
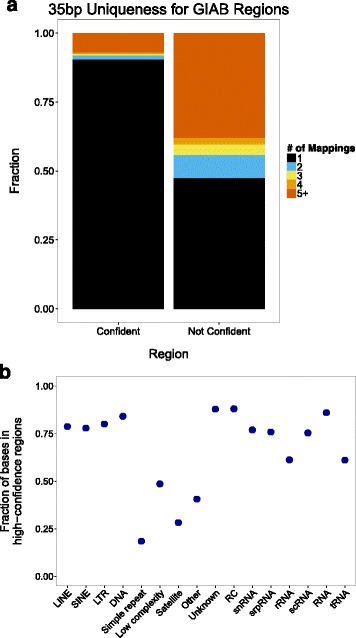
**a** The number of sites in the genome where each 35 bp sequence appears for Genome in a Bottle high-confidence and low-confidence regions. **b** The fraction of each RepeatMasker repeat class in high-confidence regions

The repetitive sequences in the reference assembly frequently cause difficulties in short-read alignment since a sequence read from a repeated region could align with equal probability to multiple locations. In these situations, results vary by aligner (and chosen alignment parameters); the read will be either: (1) placed randomly in any one of the equally best locations; (2) placed in all possible locations; or (3) not aligned at all. Aligning to repetitive sequences is particularly problematic if the patient’s genome contains a variant in one copy of a repeated sequence but not in other copies. In this case, misaligned sequence reads can create false positive or false negative variant calls, which could have clinical significance. Clearly, less repetitive regions (that is, more unique sequence) allow for improved alignments and thus improved variant calls. Therefore, we examined the uniqueness of the sequences in high and low confidence regions. We found that 90.6 % of 35 bp sequences in high-confidence regions are unique to one location in the genome compared to 47.5 % of 35 bp sequences in low-confidence regions (Fig. 3a). Further, we evaluated the fraction of each RepeatMasker repeat class in high-confidence regions. Many classes of repeats – particularly low complexity (48.6 %), simple repeats (18.5 %), and microsatellites (28.3 %) – are infrequently seen in high-confidence regions (Fig. 3b). These repetitive regions are depleted in the current high-confidence regions because regions with low mapping quality or long repeats and segmental duplications are explicitly excluded to form conservative high-confidence calls. More work is needed to form high-confidence calls in these regions.

### Characterizing ClinVar pathogenic variants

**Fig. 4:**
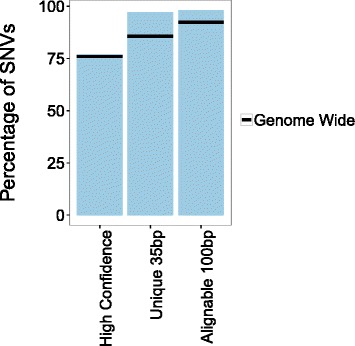
Bar graphs displaying the fraction of ClinVar pathogenic or likely pathogenic SNVs in high-confidence regions, unique sequences (35 bp), and alignable sequences (100 bp). The black line represents the genome-wide value

To understand how these analyses would be used in practice, we characterized the genomic context of all pathogenic and likely pathogenic ClinVar SNVs, whether they were in NA12878 or not. 77.14 % of the pathogenic or likely pathogenic SNVs currently in ClinVar fall within high-confidence regions, 97.17 % are at the start of a 35 bp sequence that is only present once in hg19, and 98.11 % are at the start of a 100 bp sequence that is only present once in hg19 (with up to two mismatches). These values are even higher for likely pathogenic or pathogenic SNVs with > = level 2 ClinVar review status (Table 4) and are higher than genome-wide averages (Fig. 4).

**Table 4:** Genomic context of ClinVar (likely) pathogenic SNVs

	n	%
Total likely pathogenic or pathogenic SNVs	15,735	
Likely pathogenic or pathogenic SNVs in high-confidence regions	12,138	77.14
Likely pathogenic or pathogenic SNVs that start a 35 bp unique sequence^a^	15,289	97.17
Likely pathogenic or pathogenic SNVs that start a 100 bp alignable sequence^b^	15,438	98.11
Total likely pathogenic or pathogenic SNVs with > = level 2 ClinVar review status [32]	1,212	
Likely pathogenic or pathogenic SNVs with > = level 2 ClinVar review status in high-confidence regions	998	82.34
Likely pathogenic or pathogenic SNVs with > = level 2 ClinVar review status that start a 35 bp unique sequence^a^	1,190	98.18
Likely pathogenic or pathogenic SNVs with > = level 2 ClinVar review status that start a 100 bp alignable sequence^b^	1,195	98.60

^a^The 35 bp sequence that starts at the SNV’s genomic loci is only present once in the whole reference genome (hg19)

^b^The 100 bp sequence (with up to two mismatches) that starts at the SNV’s genomic loci is only present once in the whole reference genome (hg19)

**Fig. 5:**
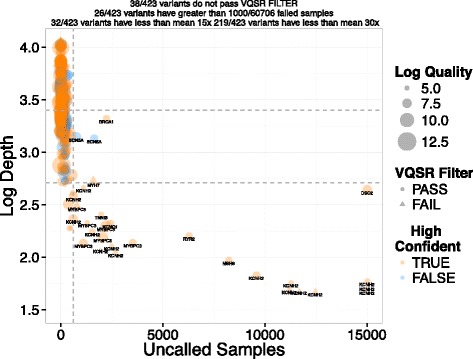
ClinVar variants within ACMG genes in the ExAC database. Depth of coverage in log_2_ space versus the number of samples that were unable to be called for that variant. The size of the points is relative to quality scores from GATK during joint calling. Orange indicates that the variant is in a high-confidence NA12878 region while blue is considered to be in low confidence. Triangles highlight variants that failed VQSR filtering

Subsequently, we looked at the distribution of ClinVar pathogenic variants within ACMG genes from 60,706 exomes in ExAC (Additional file 5: Table S4) [43]. Of the 5,146 positions classified as pathogenic, 423 were identified in this cohort. Several technical and ultimately clinical observations emerge. From the technical standpoint, a substantial number of variants were in low confidence regions (100), failed VQSR (38), had dramatically low coverage (32 were covered by less than 15 reads on average) or had suspiciously high coverage (three were covered by more than 100 reads on average) indicative of compression tracks within the reference. We examined the number of samples with uncalled genotypes as a function of read depth, and found that 26 of the 423 variant positions had low coverage and uncalled genotypes for over 1,000 of the 60,706 individuals (Fig. 5).

## Discussion

To better understand the clinical impact of technical aspects of genome sequencing, we used high-confidence consensus calls from a benchmark genome to characterize clinically relevant genetic variation at the gene and variant level across the genome. We characterized the high-confidence regions and examined the proportion of medically relevant genes that fall outside of high-confidence regions. 

### Disease causing variation occurs in complex regions of the genome

We report that large areas of key genes, as well as a significant proportion of known disease-causing variation, lie outside of high-confidence regions, highlighting the importance of technical accuracy in benchmarking clinical genomics. While less than 1,000 genes across the genome are found entirely within the high-confidence regions, it is perhaps more concerning that, of the genes we regard as most medically important – the ‘actionable’ list of 56 genes from the ACMG, only 82.1 % of their exonic structure is found within high-confidence regions. Indeed, the knowledge that nearly one fifth of each gene, for which laboratory directors are recommended to provide clinical reporting for every patient undergoing clinical exome or genome sequencing, would not reach consensus across different chemistries and pipelines, is sobering. But it is a call to arms for those interested in clinical grade technical accuracy for genome sequencing. We hope by highlighting and scrutinizing the challenging areas of the genome, we can optimize our pipelines for greater consensus and, at the very least, provide transparency regarding our confidence level in every call. In contrast with the lack of immediate personal implication of a false call in a discovery cohort study, a false call on a clinical report could have immediate detrimental consequences in the life of an individual, family, or disease community.

### False negative and false positive variants may have clinical impact

Our analysis revealed false negatives and false positives in both WES and WGS. For exome sequencing, many of false negatives were due to low or no coverage, which emphasizes the importance of choosing a sequencing platform that adequately covers all medically-relevant genomic regions [33]. Most false negatives from WGS resulted from overly aggressive filtering.

In one example of a false positive from our systematic error call set, one sequencing chemistry and one pipeline called a recognized, pathogenic frameshift deletion in *BRCA2*. Pathogenic variants in the BRCA genes are implicated in hereditary breast and ovarian cancer syndrome (http://www.ncbi.nlm.nih.gov/books/NBK1247/). The variant, rs80359760, is currently categorized in ClinVar as pathogenic/likely pathogenic based on several entries from the Breast Cancer Information Core, the Sharing Clinical Reports Project, and the literature (http://www.ncbi.nlm.nih.gov/clinvar/variation/52831/). Based on GIAB’s consensus sequence, this variant is known to be a false positive call for this patient. However, it might be reported to another patient as an incidental finding, and one with evidence for pathogenicity that might even lead to medical action. Examples like this highlight the importance of confirmatory testing by an orthogonal method. Additionally, we hope that our analyses and the reference materials can provide helpful meta-data for bioinformatics analysis of loci such as these, since this dataset allows positions with systematic biases and medically relevant annotations in public databases to be identified [44, 45].

### Analytical choices impact variant calls

Our findings highlight the influence of informatic choices upon the final variant calls. For example, the newest human reference GRCh38 employs alternate contigs, encompassing a more accurate but complex representation of normal human variation. To maximize the benefit from this significant advance requires the development of mapping, variant calling, and variant comparison [46] software that recognizes complex variation (for example, *SERPINA1* variation corresponding to an alternate locus in GRCh38, see Results) [47]. Additionally, the choice of ethnicity-specific reference genomes has been shown to impact the sensitivity of variant calling [19]. Furthermore, differences within the annotation schema employed may also influence the clinical impact of the call set [48]. Within the ACMG 56 genes in the NA12878 true positive confident call set, there were five variants that were annotated differently by one of the three gene models employed (see Additional file 6: Table S5). For example, the voltage-gated sodium channel, *SCN5A*, is associated with dilated cardiomyopathy and long QT syndromes and  displays a complex developmentally-regulated pattern of multiple splice isoforms [49]. Though the common variant rs6599230 is unlikely to be of functional significance, it was annotated as a synonymous variant p.A29A using Refgene and Gencode transcript models, and alternately annotated as a non-synonymous variant p.Q32R with a UCSC Knowngene transcript model. Each of these annotations is a true and accurate representation, each corresponding to a different splice isoform and supported by either computationally-predicted or manually-curated transcript data. However, among the multiplicity of variants, it is not clear which (or all) of these should be displayed to the ordering clinician for the purposes of clinical decision-making. Disease domain specific expertise and standardization efforts, such as those already in process by the ClinGen Resource (http://clinicalgenome.org/) will prove necessary to develop the most clinically appropriate gene models or transcripts for a particular gene. Additionally, emerging resources such as the Genotype-Tissue Expression (GTEx) project may provide relevant information for deconvoluting the isoform specific mutations in the tissue of interest.

## Conclusions

Using the reference materials developed by the Genome in a Bottle Consortium, we show that the predictive characteristics of WES and WGS for any given variant appear to depend on the genomic region, the class of variant, and the informatic tools employed. We discuss false positive and questionable variant calls from these reference materials that could significantly impact clinical care. Thus, the discussion of the technical aspects of clinical sequencing, and the continued development of reference materials to characterize more challenging parts of the genome, are critical steps toward enabling a better understanding of the predictive and technical characteristics of these tests.

## Additional files

Additional file 1: Table S1. Additional exome sequencing data for NA12878. (XLSX 8 kb) Additional file 2: Table S2. The impact of filters on false positive and true positives in the high-confidence regions of the whole genome for Platypus WGS. (XLSX 52 kb) Additional file 3: Figure S1. NCBI GeT-RM Browser visualization of alignments around a series of false positive variant calls resulting from alignment of paralogous sequence that is not in the GRCh37 reference assembly but is in a GRCh38 ALT sequence. (**a**) Alignment of Ion Torrent reads containing the series of FP SNPs. (**b**) Overview, including alignment of GRC Curation Issue HG-1930, marked with a red asterisk. (**c**) BLAST search of region revealing the same series of SNPs in ALT_REF_LOCI_1 HSCHR14_7_CTG1, which is an alternate locus in the new GRCh38 reference assembly. (DOCX 2 mb) Additional file 4: Table S3. Sites with systematic errors prior to filtering that are present in ClinVar. (XLSX 40 kb) Additional file 5: Table S4. A listing of pathogenic variants from ClinVar present in ExAC. (TXT 602 kb) Additional file 6: Table S5. Multiple annotations with transcript evidence for five variants in ACMG genes detected in the NA12878 consensus standard dataset. (XLSX 39 kb)
