# Supplementary material for: Medical implications of technical accuracy in genome sequencing
Source: Genome Med. 2016 Mar 2;8:24. doi: 10.1186/s13073-016-0269-0 (PMC4774017; doi:10.1186/s13073-016-0269-0)
Supplement: Additional file 3: Figure S1. — NCBI GeT-RM Browser visualization of alignments around a series of false positive variant calls resulting from alignment of paralogous sequence that is not in the GRCh37 reference assembly but is in a GRCh38 ALT sequence. (a) Alignment of Ion Torrent reads containing the series of FP SNPs. (b) Overview, including alignment of GRC Curation Issue HG-1930, marked with a red asterisk. (c) BLAST search of region revealing the same series of SNPs in ALT_REF_LOCI_1 HSCHR14_7_CTG1, which is an alternate locus in the new GRCh38 reference assembly. (DOCX 2 mb) [file 13073_2016_269_MOESM3_ESM.docx]

**Supplemental Figure 1**

A.
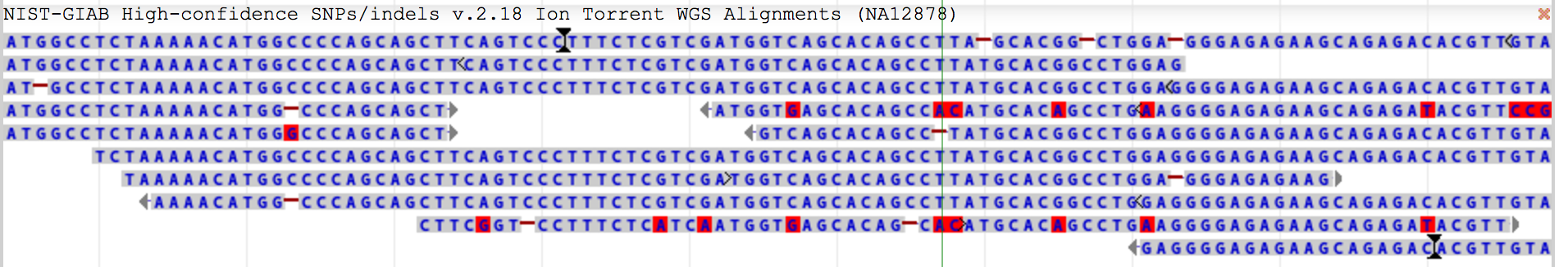


B.


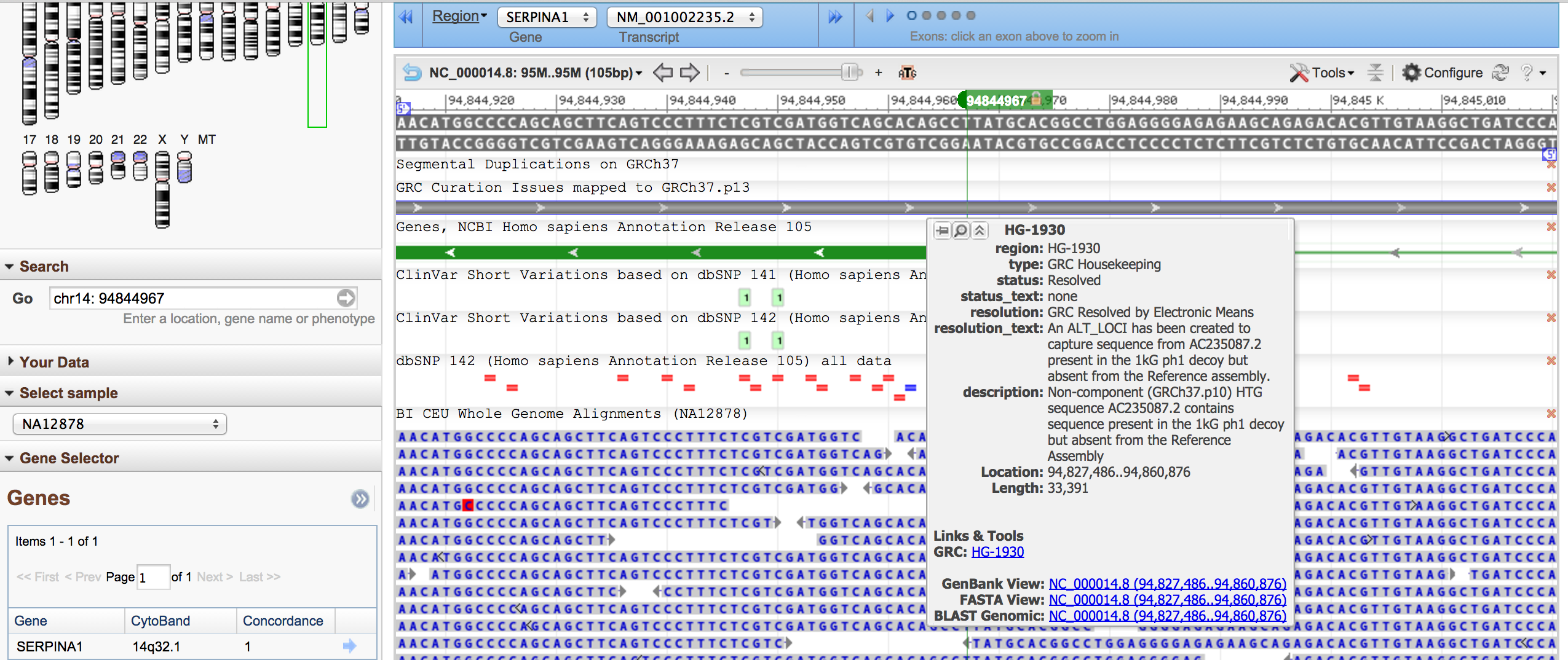


*

C.
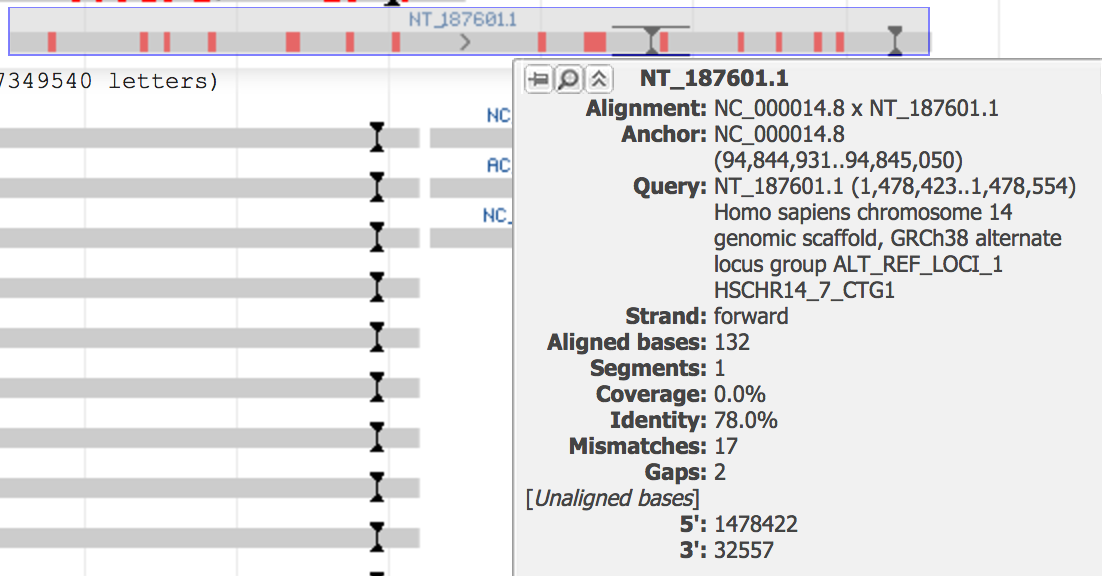


Supplemental Figure 1: NCBI GeT-RM Browser visualization of alignments around a series of false positive variant calls resulting from alignment of paralogous sequence that is not in the GRCh37 reference assembly but is in a GRCh38 ALT sequence. (a) Alignment of Ion Torrent reads containing the series of FP SNPs. (b) Overview, including alignment of GRC Curation Issue HG-1930, marked with a red asterisk. (c) BLAST search of region revealing the same series of SNPs in ALT_REF_LOCI_1 HSCHR14_7_CTG1, which is an alternate locus in the new GRCh38 reference assembly.
